# Management of anterior cruciate ligament revision in adults: the 2022 ESSKA consensus part III—indications for different clinical scenarios using the RAND/UCLA appropriateness method

**DOI:** 10.1007/s00167-023-07401-3

**Published:** 2023-05-03

**Authors:** Thomas Tischer, Luca Andriolo, Philippe Beaufils, Sufian S. Ahmad, Corrado Bait, Marco Bonomo, Etienne Cavaignac, Riccardo Cristiani, Matthias J. Feucht, Markas Fiodorovas, Alberto Grassi, Gijs Helmerhorst, Christian Hoser, Mustafa Karahan, George Komnos, Koen Carl Lagae, Vincenzo Madonna, Edoardo Monaco, Juan Carlos Monllau, Matthieu Ollivier, Mikko Ovaska, Wolf Petersen, Tomasz Piontek, James Robinson, Kristian Samuelsson, Sven Scheffler, Bertrand Sonnery-Cottet, Giuseppe Filardo, Vincenzo Condello

**Affiliations:** 1 Department of Orthopaedic and Trauma Surgery, Waldkrankenhaus, Erlangen, Germany; 2 Department of Orthopaedic Surgery, University Medicine Rostock, Rostock, Germany; 3 Clinica Ortopedica e Traumatologica 2, IRCCS Istituto Ortopedico Rizzoli, Via Pupilli 1, 40136 Bologna, Italy; 4 ESSKA Consensus Projects, Versailles, France; 5 Department of Orthopaedic Surgery, Medical School of Hannover MHH, Annastift Hospital, Hannover, Germany; 6 Joint Preservation Surgery and Sport Medicine Unit, Villa Aprica Clinical Institute, Como, Italy; 7 Dipartimento di Ortopedia e Traumatologia, IRCCS Ospedale Sacro Cuore don Calabria, Negrar, VR Italy; 8 Musculoskeletal Institute, Hôpital Pierre Paul Riquet, CHU Toulouse, Toulouse, France; 9 Capio Artro Clinic, FIFA Medical Centre of Excellence, Valhallavägen 91, 11486 Stockholm, Sweden; 10 Department of Molecular Medicine and Surgery, Stockholm Sports Trauma Research Center, Karolinska Institutet, Stockholm, Sweden; 11 Department of Orthopaedic Surgery Paulinenhilfe, Diakonie Klinikum, Stuttgart, Germany; 12 Department of Orthopaedics and Trauma Surgery, Medical Center, Faculty of Medicine, Albert-Ludwigs-University of Freiburg, Freiburg, Germany; 13 Northway Medical Center, Klaipeda, Lithuania; 14 Department of Orthopaedic Surgery, Amsterdam University Medical Centre (AUMC) and Flevoziekenhuis, Amsterdam, The Netherlands; 15 Praxisgemeinschaft Gelenkpunkt, Innsbruck, Austria; 16 MAA Acıbadem University, Istanbul, Turkey; 17 Orthopaedic Department, University Hospital of Larisa, Larisa, Greece; 18 Knee and Sports Surgery, Knee Department, Monica Hospitals, Antwerp, Belgium; 19 Hopital Delta, Brussels, Belgium; 20 Physioclinic, Milan, Italy; 21 Joint Preservation and Reconstructive Surgery and Sports Medicine Unit, Humanitas Castelli Clinic, Bergamo, Italy; 22 Orthopaedic Unit, University of Rome La Sapienza, Sant’Andrea Hospital, Rome, Italy; 23 Department of Orthopaedics and Traumatology, Parc de Salut Mar, ICATME-Hospital Universitari Dexeus, Universitat Autònoma de Barcelona (UAB), Barcelona, Spain; 24 Institut for Movement and Locomotion, Hôpital Sainte Marguerite, UMR 7287, Aix-Marseille Université et CNRS, Marseille, France; 25 Lower Extremity Unit, Pihlajalinna Pikkuhuopalahti, Helsinki, Finland; 26 Martin Luther Hospital, Berlin, Germany; 27 Rehasport, Spine Disorders and Pediatric Orthopaedic Department, University of Medical Sciences, Poznań, Poland; 28 Knee Specialists, Bristol, UK; 29 Department of Orthopaedics, Institute of Clinical Sciences, Sahlgrenska Academy, University of Gothenburg, Gothenburg, Sweden; 30 Department of Orthopaedics, Sahlgrenska University Hospital, Mölndal, Sweden; 31 Sporthopaedicum Berlin, Berlin, Germany; 32 Centre Orthopédique Santy, FIFA medical centre of Excellence, Hôpital Privé Jean Mermoz, Ramsay, Lyon, France; 33 Applied and Translational Research (ATR) Center, IRCCS Istituto Ortopedico Rizzoli, Bologna, Italy; 34 Faculty of Biomedical Sciences, Università della Svizzera Italiana, Lugano, Switzerland; 35 Service of Orthopaedics and Traumatology, Department of Surgery, EOC, Lugano, Switzerland

**Keywords:** Anterior cruciate ligament, Revision, Consensus, Knee, Guidelines

## Abstract

**Purpose:**

The aim of the ESSKA 2022 consensus Part III was to develop patient-focused, contemporary, evidence-based, guidelines on the indications for revision anterior cruciate ligament surgery (ACLRev).

**Methods:**

The RAND/UCLA Appropriateness Method (RAM) was used to provide recommendations on the appropriateness of surgical treatment versus conservative treatment in different clinical scenarios based on current scientific evidence in conjunction with expert opinion. A core panel defined the clinical scenarios with a moderator and then guided a panel of 17 voting experts through the RAM tasks. Through a two-step voting process, the panel established a consensus as to the appropriateness of ACLRev for each scenario based on a nine-point Likert scale (in which a score in the range 1–3 was considered ‘inappropriate’, 4–6 ‘uncertain’, and 7–9 ‘appropriate’).

**Results:**

The criteria used to define the scenarios were: age (18–35 years vs 36–50 years vs 51–60 years), sports activity and expectation (Tegner 0–3 vs 4–6 vs 7–10), instability symptoms (yes vs no), meniscus status (functional vs repairable vs non-functional meniscus), and osteoarthritis (OA) (Kellgren–Lawrence [KL] grade 0–I–II vs grade III). Based on these variables, a set of 108 clinical scenarios was developed. ACLRev was considered appropriate in 58%, inappropriate in 12% (meaning conservative treatment is indicated), and uncertain in 30%. Experts considered ACLRev appropriate for patients with instability symptoms, aged ≤ 50 years, regardless of sports activity level, meniscus status, and OA grade. Results were much more controversial in patients without instability symptoms, while higher inappropriateness was related to scenarios with older age (51–60 years), low sporting expectation, non-functional meniscus, and knee OA (KL III).

**Conclusion:**

This expert consensus establishes guidelines as to the appropriateness of ACLRev based on defined criteria and provides a useful reference for clinical practice in determining treatment indications.

**Level of evidence:**

II.

## Introduction

The anterior cruciate ligament (ACL) plays a key role in knee stability, and ACL injury may be responsible for severe functional impairment [17]. ACL tears are common and affect mostly young, active patients [2], in which ACL reconstruction (ACLR) is often considered the first-choice treatment with generally good results reported [5]. Nevertheless, a 6.2% graft failure rate (0–13.4%) and an overall failure rate of 11.9% (3.2–27%) at 10 years are reported [3], making revision ACL surgery (ACLRev) an increasingly performed orthopedic procedure. The goals of ACLRev are to restore joint stability, allow a safe return to sport and to reduce deleterious forces that may lead to the early osteoarthritic (OA) changes observed in unstable knees [10]. To improve diagnostics, preoperative planning, and surgical strategy in ACLRev, parts one and two of the European Society for Sports Traumatology, Knee Surgery and Arthroscopy (ESSKA) consensus formulated consensus statements [16, 18, 19]. However, the indications for surgical or conservative management of ACL graft failure are dependent on numerous factors. Despite a growing body of literature defining the parameters that influence the outcomes of ACL graft rupture and ACLRev [6, 9, 13], there is little in the way of practical, clinical guidelines to assist surgeons. There is a lack of consensus as to the appropriate indications for ACLRev versus conservative management for the varying clinical scenarios and patient presentations encountered in clinical practice.

The RAND/UCLA Appropriateness Method (RAM) is a group-consensus method developed to produce, through a highly structured approach, patient-specific recommendations combining the best available scientific evidence with the collective judgement of a panel of experts. The methodology was developed in the 1980s [8], and since, has been used extensively for assessing the appropriateness of medical and surgical procedures (e.g. vertebral fragility fractures, chondral and osteochondral scaffolds, coronary angiography, carotid endarterectomy, etc.) [14]. Several studies support the reliability, internal consistency and clinical validity of RAM-based recommendations [14]. The method allows for both confidential ratings as well as group discussion, it has moderate to excellent reproducibility as determined by different panelists for “appropriate” and “inappropriate” care and it has an acceptable predictive validity for a recommendation supported by RCTs [14]. The presence of a core panel and a moderator limits potential relational biases due to face-to-face confrontation with highly opinionated individuals dominating discussion [14]. The RAM group consensus is thus able to provide specific, clear recommendations as to the appropriateness of indications for treatment related to the supporting scientific evidence.

ESSKA set up a RAM expert consensus process with the aim of developing patient-focused, contemporary, evidence-based, guidelines on the indications for ACLRev for different clinical scenarios.

## Material and methods

### Consensus design

The RAM was used to develop recommendations on the appropriateness of ACLRev in adult patients affected by primary ACLR graft failure [8]. ACL revision was defined as “all surgical procedures involving replacement of the ACL graft with a new graft”. For the purpose of this consensus, failure has been defined by abnormal knee function associated with a previous primary reconstruction. This could be due to graft failure itself with abnormal laxity (IKDC C/D) or failure to recreate a functional knee according to the expected outcome [18]. The RAM process involved a core panel including a moderator and an expert panel. The core panel (FG, TT, PB, LA, VC) defined the scenarios of the RAM and guided the expert panel through the RAM tasks. The expert panel, composed of 17 voting members selected based on their expertise and at the same time ensuring geographical representation, used the data provided by the core panel to come to a consensus. The members were selected based on their scientific and clinical expertise in ACLRev while ensuring the geographical representation of ESSKA European members.

### Clinical scenarios development

The RAM process was preceded by an extensive literature review undertaken by the steering group of the parallel “Formal Consensus Project”, set up by ESSKA, on the diagnosis, preoperative planning, and surgical strategy for ACLRev (parts I and II). This literature review ensured that panelists had access to the body of evidence for the rating procedure and was used by the core panel to develop the consensus scenarios. These clinical scenarios were presented in the form of a matrix detailing demographic data, characteristics of the joint and association with combined lesions. These factors were based on literature evidence suggesting a correlation with the clinical outcomes after surgery, potentially influencing the appropriateness of the procedure:

- Age (18–35 years old vs 36–50 years old vs 51–60 years old)
- Sports participation expectation (tegner activity level 0–3 vs 4–6 vs 7–10)
- Instability symptoms (yes vs no)
- Meniscus status (functional meniscus vs repairable meniscus vs non-functional meniscus)
- OA (Kellgren–Lawrence [KL] [11] grade 0–I–II vs grade III)

For the scenarios, it was assumed that patients lacked gross osseous malalignment (varus/valgus within 5°, tibial sagittal slope less than 12°), had no additional ligamentous injuries and did not have advanced OA (KL IV).

Sport expectation participation was considered as not only the sporting level previously practiced by the patient, but also the desired activity level following surgical reconstruction. Instability was defined as a functional symptom (subjective) where “an abnormal dynamic joint motion occurs in response to the complex, high-magnitude loads encountered during activities of daily living and sport activities.” On the other hand, pathological laxity (objective) was defined as “an increased passive response of a joint to an externally applied force or torque in biomechanical terms”. Meniscus status was classified as “functional meniscus” in the case of no meniscal lesion or limited lesions requiring meniscectomy but not compromising meniscal function; “repairable meniscus” was represented by a lesion suitable for meniscal suture or repair; “non-functional meniscus” included patients with previous meniscectomy or large irreparable meniscal lesions.

Patients aged over 60 were excluded in view of the lack of evidence available on the outcomes of ACLRev in this age group. Similarly, patients with advanced OA (KL IV) were also excluded. However, the indications for ACLRev in patients over the age of 60 and in patients with advanced OA (KL IV) were addressed in two further questions following the ESSKA formal consensus process. Although ACLRev can be combined with a high tibial osteotomy to improve symptomatic ACL instability in patients with OA knee [20], realignment procedures were excluded from the consensus, as it was felt that the inclusion of malalignment would have created too many variables for consideration, Similarly, the presence of combined ligamentous injury (e.g. posterior cruciate ligament, collateral ligaments, posterolateral corner, etc.) was also excluded from the clinical scenarios. Smoking status, BMI, gender and the interval between ACL graft rupture and reconstruction were also excluded from the scenarios in view of a lack of evidence suggesting these factors can influence outcomes.

**Fig. 1:**
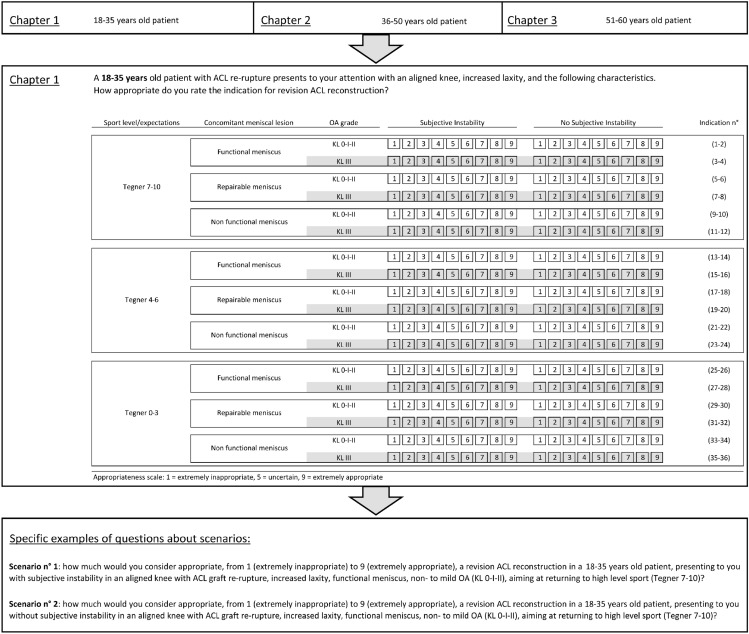
Example of the clinical scenarios presented to the voting panelists. Chapter 1 (18–35 years old patients). Two specific scenarios are shown in detail

Based on the five clinical variables considered most relevant by the core expert panel, a set of 108 clinical scenarios were produced. Panelists were asked to individually assess the appropriateness of the indication for ACLRev for each of the scenarios. The scenarios were grouped into three “chapters” based on patient age. (Fig. 1). The scenarios were presented to the voting experts in the form of a question: “A … years old patient with ACL re-rupture presents to your attention with an aligned knee, increased laxity, and the following characteristics. How appropriate do you rate the indication for ACLRev?”.

### Consensus process

The appropriateness of the indication for surgical treatment in each of the different scenarios was rated in two rounds. The two-round RAM process is designed to identify whether discrepant ratings are due to real clinical disagreement over the use of the procedure ("real" disagreement) or to fatigue or misunderstanding ("artefactual" disagreement) [8].

In the first round, the expert panel received the clinical scenarios by email and was asked to rate the appropriateness of the indication for ACLRev. According to the RAM [8], each panelist ranked, independently from the other panelists, the appropriateness for each scenario on a nine-point Likert scale, in which a score in the range 1–3 is considered ‘inappropriate’, 4–6 ‘uncertain’, and 7–9 ‘appropriate’. They were invited to consider the synthesized evidence from the literature review provided by the core panel overseeing the consensus process. The expert panelists were asked to discount the cost of the procedure in rating the appropriateness of the scenarios.

In the second round, the experts’ panel and the core panel met under the leadership of a moderator. Each panelist received an individualized document showing their round one rating and the distribution of all the expert group’s first-round ratings. During the meeting, panelists discussed the ratings, focusing on areas of disagreement. The opportunity was given to modify the original list of indications and/or definitions if desired. The panel was not forced to reach a consensus and, after discussing each chapter of scenarios, experts individually re-rated the appropriateness of the indication for ACLRev for each scenario [8].

### Data analysis

The final nine-point Likert-scale scores of each expert were then pooled to generate a median appropriateness score for each scenario. In addition, according to the RAM, the presence of “disagreement” was calculated according to the following definition: at least six panelists rated the indication in the 1–3 region and at least six panelists rated it in the 7–9 region [8]. Finally, the indication for ACLRev for each clinical scenario was classified:

- “appropriate”: median score of ≥ 7 without disagreement
- “inappropriate”: median vote of ≤ 3 without disagreement

The rating of “Inappropriate” indicated that the expert consensus favored conservative management rather than ACLRev for that specific clinical scenario.

A scenario receiving a score between 4 and 6, or a scenario with disagreement, was classified as “uncertain”. An “uncertain” recommendation can reflect either the ambiguous state of currently available evidence or equivocal appropriateness either due to a moderately unfavorable risk profile or to limited efficacy. The ‘uncertain’ classification is not intended to be a negative recommendation or to preclude the use of the treatment for the specific scenario based on surgeon–patient shared decision making in the context of individual circumstances, co-morbidities, and preferences.

## Results

**Fig. 2:**
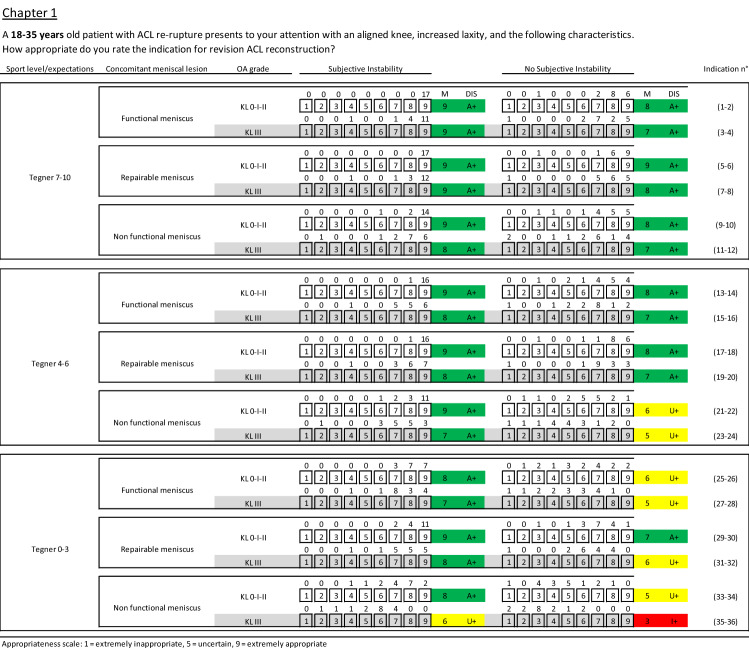
Clinical scenarios for the age range 18–35 (chapter 1). M median value, DIS disagreement, A appropriate, U uncertain, I inappropriate, + without disagreement, − with disagreement, green appropriate scenarios, yellow uncertain scenarios, red inappropriate scenarios

**Fig. 3:**
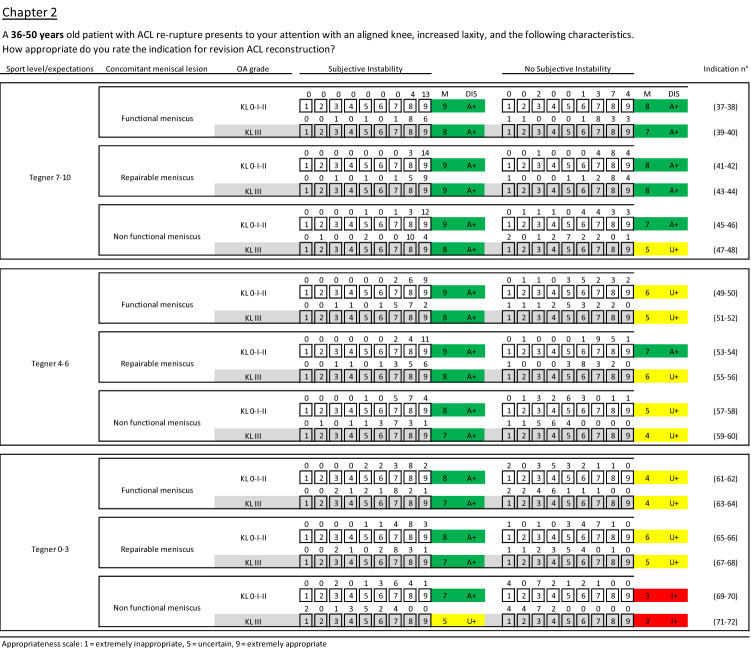
Clinical scenarios for the age range 36–50 (chapter 2). M median value, DIS disagreement, A appropriate, U uncertain, I inappropriate, + without disagreement, − with disagreement, green appropriate scenarios, yellow uncertain scenarios, red inappropriate scenarios

**Fig. 4:**
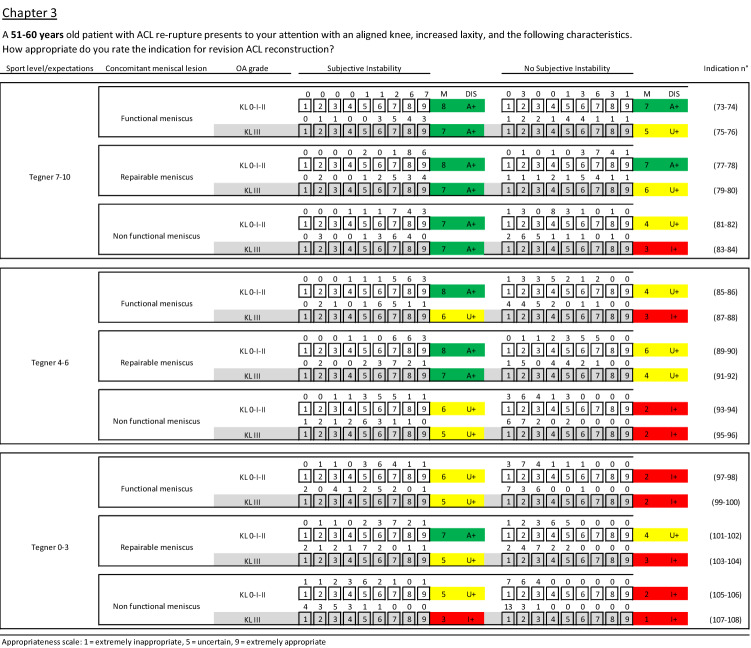
Clinical scenarios for the age range 51–60 (chapter 3). M median value, DIS disagreement, A appropriate, U uncertain, I inappropriate, + without disagreement, − with disagreement, green appropriate scenarios, yellow uncertain scenarios, red inappropriate scenarios

Details of experts’ ratings with median, agreement value and recommendation for each clinical scenario are reported in Figs. 2, 3, and 4. Following the two voting rounds, there was an agreement for 76 (70%) of the scenarios: in 63 scenarios (58%) the indication for ACLRev was considered appropriate without disagreement, in 13 (12%) inappropriate without disagreement, and in 32 (30%) scenarios the indication was uncertain.

### Appropriateness, inappropriateness, and uncertain areas

#### Patients with instability symptoms

Experts considered ACLRev appropriate for every patient with instability symptoms aged ≤ 50 years, regardless of sport activity level, meniscus status, and OA grade (α). The exception was for the scenarios in which patients had low sports activity scores, non-functional meniscus, and OA KL III (scenarios no. 35 and 71).

If instability symptoms were present in patients over 50 years old, ACLRev was indicated in those with high sports activity scores (Tegner 7–10, β). For lower sports activity levels (Tegner 4–6), the only scenarios where surgery was considered appropriate were for patients with functional meniscus and without moderate OA (indication no. 85), and patients with repairable menisci regardless of OA grade (indications no. 89 and 91). For Tegner scores 0–3, the only scenario where ACLRev was considered appropriate was a repairable meniscus without OA (indication no. 101), whereas revision surgery was considered inappropriate for patients with OA KL III and with non-functional meniscus (scenario no. 107). The remaining scenarios were evaluated as uncertain (γ).

#### Patients without instability symptoms

The indications for ACLRev were much more controversial in patients without instability symptoms. For these patients, more significance was given to age and participation in sports. For patients with Tegner scores 7–10, ACLRev was indicated for all patients ≤ 50 years old (δ) with the only exception being a patient aged 36–50 with non-functional meniscus and moderate OA (scenario no. 48). There was agreement on the indications for ACLRev in 18–35 years old, with a Tegner score of 4–6, with functional or repairable meniscus (ε). However, for the remaining scenarios (older patients and/or lower levels of sports participation), there was little agreement on the indication for ACLRev except in patients with repairable meniscus and no/mild OA (patients aged 18–35 and Tegner score 1–3, patients aged 36–50 and Tegner score 4–6 and patients aged 51–60 with Tegner score 7–9, scenarios no. 30, 54, and 78, respectively) and for a patient aged 51–60, with Tegner score 7–9, a functional meniscus and OA KL 0–I–II (scenario no. 74).

ACLRev was considered inappropriate (meaning conservative management is indicated) in patients without instability in patients with a non-functional meniscus with or without OA participating in lower levels of sport (ζ) or those of older age (η), or for older patients with low or intermediate levels of sports participation (θ). The indication for ACLRev was considered uncertain for almost half of the scenarios where there were no instability symptoms (ι).

### Appropriateness changes within parameters

The analysis demonstrated that certain factors were more influential in determining the appropriateness of ACLRev. The presence of instability symptoms influenced 34 out of 54 treatment indications (63.0%). Sports activity level was also a key discriminating factor: having as a reference point the treatment indication in the 36 scenarios with Tegner 7–10, treatment indication in the Tegner 4–6 group changed (toward uncertain or inappropriate) in 13 scenarios and in the Tegner 0–3 group in 23 scenarios, for a total of 36 changes out of 72 scenarios (50.0%). Age changed the appropriateness in 28 of the 72 scenarios differing only for age (38.9%), meniscus status changed appropriateness in 25 of the 72 scenarios differing only for this factor (34.7%), and OA changed the appropriateness in 14 of the 54 couples of scenarios differing only for KL grade (25.9%).

### Consensus results for each specific factor

**Fig. 5:**
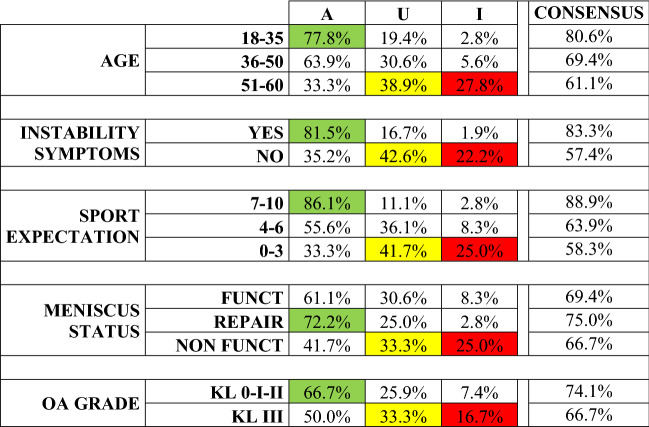
Rating of scenarios evaluated as appropriate, uncertain, or inappropriate, for each parameter considered. Green indicates the highest rates of appropriateness, yellow the most uncertain parameters, and red the highest rate of inappropriateness when considering the indication for ACLRev based on the different parameters evaluated. A appropriate, U uncertain, I inappropriate, OA osteoarthritis, KL Kellgren Lawrence, Funct functional, Repair repairable

The different patient factors evaluated in the clinical scenarios had a varying influence on the appropriateness of the indication for ACLRev (Fig. 5).

The factors which most influenced the appropriateness of the indication (86.1% of appropriate scenarios) were high sports activity level (Tegner 7–10), the presence of instability symptoms (81.5% of appropriate scenarios), and age 18–35 (77.8% of appropriate scenarios). Conversely, the parameters associated with higher inappropriateness ratings were age 51–60 (27.8% of inappropriate scenarios), low sports activity level (Tegner 0–3) and non-functional meniscus (both 25% of inappropriate scenarios). The presence of a repairable meniscal lesion was associated with a high rate of appropriateness for ACLRev (72.2%), compared to the functional meniscus (61.1%) and non-functional meniscus (41.7%). The parameter determining the lowest rates of appropriateness and inappropriateness was OA (66.7% of appropriate scenarios for OA KL 0-I-II, and 16.7% of inappropriate scenarios for OA KL III).

**Fig. 6:**
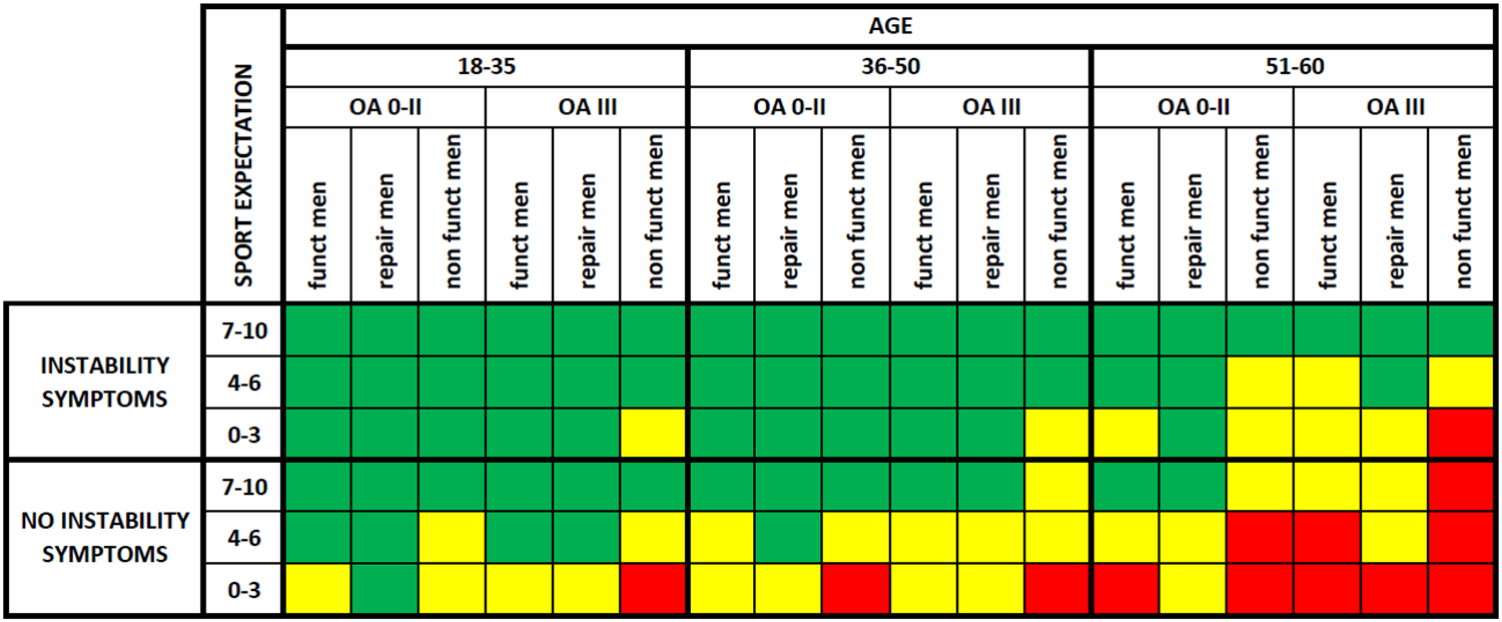
Graphic representation of the overall RAM consensus results on the appropriateness of ACLRev in adults (Green: appropriate; yellow: uncertain; red: inappropriate). OA: osteoarthritis, Funct: Functional, Repair: Repairable, Men: Meniscus

A graphic representation of the overall consensus results is shown in Fig. 6.

### Results of ESSKA formal consensus process for older age and advanced OA

*I1*: What is the indication for performing an ACLRev in people older than 60 years?

*Consensus answer*: No evidence is available on the outcomes of ACLRev in patients older than 60 years of age. However, based on the evidence available for ACL primary reconstruction, ACLRev is not contraindicated, especially in active patients with symptomatic instability and limited OA.

*Grade*: D

*I2*: Are there indications to perform ACLRev in patients with KL4?

*Consensus answer*: ACLRev can be effective in reducing activity-induced pain and instability in early OA. For advanced OA (KL 4) there is no indication to perform isolated ACLRev. Data for combined surgery are only rarely available, but high tibial osteotomy can be combined with ACLRev in special indications to improve symptomatic instability in the OA knee.

*Grade*: D

## Discussion

The main finding of this part of the ESSKA ACLRev consensus project was the considerable agreement amongst experts as to the appropriate indications for ACLRev (versus conservative management), identifying scenarios where surgery is indicated and also when surgery is not appropriate. This consensus should assist surgeons in the management of patients with primary ACL graft rupture in clinical practice.

The consensus found that the appropriate indications for ACLRev are mainly driven by participation in high-level sports activity (Tegner scores of 7–10), young age (18–35 years) and instability. The presence of a repairable meniscus lesion also increased the appropriateness of ACLRev. The presence of moderate OA (KL III) was only considered a contraindication to ACLRev in 16.7% of scenarios in which it was a factor, whereas the formal consensus reported that advanced OA (KL IV) was regarded as a contraindication for isolated ACLRev. Most uncertainties with regard to the appropriateness of ACLRev were seen when considering older people (51–60) with sports activity levels less than a Tegner score of 7 and in patients without instability symptoms. However, patients older than 50 years of age may still be considered for ACLRev if the patient wishes to return to high levels of activity and has instability symptoms and only limited OA.

Two of the four factors influencing whether ACLRev is appropriately indicated are subjective (activity level and instability symptoms), highlighting the importance of taking a thorough history as part of the detailed preoperative workup. This has also been highlighted in the ESSKA consensus diagnostics and preoperative planning section [18]. Patients’ individual expectations are very important, as well as their commitment to not only undergo revision surgery but to also comply with the necessary rehabilitation to return to desired activity levels. Feucht et al. have shown that in general the expectations after ACL graft rupture are somewhat lower than for primary reconstruction but still remain high [7].

Some important points were raised during the discussions following the rating process. Some experts considered ACLRev useful to prevent further joint degeneration [1] and were very committed to promoting the appropriateness of surgery, while others focused more on symptomatic instability and highlighted the fact that conservative therapy including intensive rehabilitation may also be a successful strategy, particularly for those without symptomatic instability, leaving revision surgery for those that fail conservative management [4, 12].

The protective role of ACLRev in terms of OA prevention remains controversial; however, there was strong expert consensus as to the benefits of ACLRev in protecting the meniscus. The presence of a repairable meniscal lesion indicated that ACLRev was appropriate for most experts. Meniscal preservation at the time of ACLRev has been suggested to prevent subsequent OA by eliminating one main contributing factor for OA development [15].

This paper has some weaknesses. Whilst the RAND/UCLA Appropriateness Method (RAM) offers the possibility to investigate treatment appropriateness in different scenarios, these are still based on an oversimplification of the patients, who present individual nuances that may not be well represented by the consensus results. Moreover, some presented scenarios derived from the consensus methodological process are uncommon and may be non-representative of clinical practice, for example very active (Tegner 7–10) elderly people (> 50 years), or repairable meniscus in older people with moderate OA. Thus, the results of this consensus should be used as broad indications on how the different factors should be considered when deciding on the most appropriate treatment, rather than strict indications. They should be related to individual patients carefully. A further limitation of this paper is the lack of clear evidence in some areas. “Uncertain” indications for ACLRev may be due to a moderately unfavorable risk profile, limited efficacy, or simply due to the lack of a sufficient literature base. Patients for whom the indication for ACLRev is uncertain should be carefully counselled and the treatment decision made in the context of individual characteristics and preferences. Scenarios where the indication for revision surgery is uncertain are an area to focus future research to improve the available evidence.

## Conclusions

This expert consensus establishes guidelines as to the appropriateness of ACLRev based on defined criteria and provides a useful reference for clinical practice in determining treatment indications.

## Scenarios groups

α: scenarios no. 1, 3, 5, 7, 9, 11, 13, 15, 17, 19, 21, 23, 25, 27, 29, 31, 33, 37, 39, 41, 43, 45, 47, 49, 51, 53, 55, 57, 59, 61, 63, 65, 67, and 69.
δ: scenarios no. 2, 4, 6, 8, 10, 12, 38, 40, 42, 44, and 46.
ε: scenarios no. 14, 16, 18, and 20.
ζ: scenarios no. 36, 70, 72, 106, and 108.
η: scenarios no. 84, 94, and 96.
θ: scenarios no. 88, 98, 100, and 104.
ι: scenarios no. 22, 24, 26, 28, 32, 34, 48, 50, 52, 56, 58, 60, 62, 64, 66, 68, 76, 80, 82, 86, 90, 92, and 102.

## Data Availability

No datasets were generated or analysed during the current study.
